# Impact of Relative Humidity on Heat Stress Responses in Early-Lactation Holstein Cows

**DOI:** 10.3390/ani15111503

**Published:** 2025-05-22

**Authors:** Janghoon Jo, Honggu Lee

**Affiliations:** 1 Department of Animal Science, Purdue University, West Lafayette, IN 47907, USA; jo77@purdue.edu; 2 Department of Animal Science and Technology, Sanghuh College of Life Sciences, Konkuk University, Seoul 05029, Republic of Korea

**Keywords:** dairy cow, heat stress, milk energy, relative humidity

## Abstract

High relative humidity can increase the risk of heat stress in dairy cows, but its effects in humid continental climates are not well defined. This study evaluated how humidity levels influence dry matter intake, milk yield and composition, physiological response, blood hematology, metabolites, hormones, and heat shock protein gene expression in hair follicles. Sixteen multiparous early-lactating Holstein cows were assigned to four groups in a 2 × 2 factorial design with two temperatures (25 °C and 31 °C) and two humidity levels (50% (LRH) and 80% (HRH)). All cows were housed in climate-controlled chambers. Compared with LRH, HRH at both temperatures reduced dry matter intake, milk yield, milk fat, energy-corrected milk, and 3.5% fat-corrected milk. HRH also increased rectal temperature, heart rate, glucose, blood urea nitrogen, cortisol, and heat shock protein 90 in hair follicles. These results show that HRH, even at moderate temperature, affects dairy cow performance and alters physiological and metabolic functions. These findings emphasize the importance of precise management strategies to reduce the effects of humid heat stress on dairy performance. A comprehensive understanding of these responses is crucial for developing effective, sustainable solutions to ensure the viability of the dairy industry under increasing temperature and humidity.

## Simple Summary

This study focuses on the significant impact of relative humidity on heat stress in Holstein cows during early lactation, a critical period for dairy production. The findings demonstrate that elevated relative humidity levels reduce dry matter intake, milk yield, and milk composition, while increasing rectal temperature, heart rate, glucose, and cortisol. Additionally, higher relative humidity levels upregulate heat shock protein 90 gene expression in hair follicles, suggesting a genetic response to heat stress. These results emphasize the crucial role of relative humidity in exacerbating heat stress, highlighting the need for effective management strategies to mitigate its negative effects. Understanding how relative humidity influences dairy cow performance is essential for developing solutions that protect productivity and animal welfare. As global temperatures and relative humidity levels increase, addressing the challenges of humid heat stress is vital for the sustainability of the dairy industry and ensuring its resilience in a changing climate.

## 1. Introduction

Heat is transferred through sensible (non-evaporative) and latent (evaporative) mechanisms. Conduction, convection, and radiation are influenced by temperature gradients between the body and its environment, while evaporation is primarily driven by the vapor pressure gradient [1]. As the relative humidity (RH) increases, the gradient is reduced, thereby decreasing the evaporative efficiency. The resulting impairment of heat dissipation increases the risk of heat stress (HS). Elevated RH has also been linked to an increased incidence of several diseases, including cardiovascular and gastrointestinal diseases, and high rates of morbidity and mortality [2]. However, evidence of the health impacts of elevated RH is inconsistent, as a study reported minimal or no association with heat-related mortality [3]. It has been recommended that the health effects of temperature and RH be examined independently [4]. Exploring the specific impact of high RH, independent of high temperatures, is essential for accurately assessing its role in heat-related health outcomes. Research has shown that RH levels above 70% can significantly influence physiological responses, with these effects further intensifying under rising temperatures [5].

Dairy cows are homeothermic animals that generate considerable heat through basal metabolism, lactation, and genetic regulation to maintain body temperature through various heat-balancing mechanisms. HS has been shown to impair productivity, compromise health, and increase mortality [6,7], leading to substantial financial losses for the livestock industry, with the United States dairy industry facing an annual loss of approximately USD 1 billion [8]. Effective thermoregulation in high-producing cows depends significantly on sweating as a primary heat dissipation mechanism [9]. However, elevated RH can hinder evaporation [10], highlighting the need to understand how rising ambient temperature and varying RH levels affect heat transfer. HS occurs when the capacity to dissipate heat is surpassed by internal thermogenesis and environmental heat accumulation [11].

Therefore, this study was designed to assess the impact of humidity-induced HS on productivity, physiological responses, blood and milk parameters—hematology, metabolites, hormones, and composition—and heat shock protein (HSP) gene expression in dairy cows. By examining how different RH levels modulate HS responses, the research seeks to clarify the effects on key production metrics, such as dry matter intake (DMI) and milk yield. The study findings are expected to enhance the understanding of the physiological mechanisms underlying humidity-related stress, and to offer critical insights for improving dairy farm management practices under high-temperature conditions.

## 2. Materials and Methods

### 2.1. Experimental Design and Animals

The experimental procedure was approved by the Institutional Animal Care and Use Committee of Konkuk National University (Approval No. KU19121). A total of 16 multiparous Holstein cows were selected from a group of 80 based on comparable parity, days in milk, and milk yield to ensure group uniformity (*n* = 4 per group). Following previous studies that used one cow per metabolic chamber for HS research [12,13,14], temperature and RH treatments were implemented under controlled conditions. The selected cows had an average parity of 2.6 ± 0.27 (*p* > 0.05), 40 ± 9 days in milk (*p* > 0.05), and a milk yield of 30 ± 1.5 kg/day (*p* > 0.05). The temperature–humidity index (THI) was calculated using the following equation, provided by Jo et al. [12]: THI = (1.8 × T_db_ + 32) − [(0.55 − 0.0055 × RH) × (1.8 × T_db_ − 26)], where T_db_ represents the dry-bulb temperature (°C) and RH denotes the relative humidity (%). Since the air velocity was negligible within the metabolic chamber, wind speed was excluded from the calculation, consistently with prior findings indicating minimal impact under similar experimental conditions in South Korea. Based on relevant HS indicators, the equation by Jo et al. [12] was considered the most suitable for our study. Before HS exposure, early-lactation Holstein cows were randomly assigned to individual metabolic chambers (3 m × 4 m × 5 m) and subjected to a 3-day adaptation period under thermoneutral conditions (22 °C, 50% RH, THI = 68), during which no treatment was administered. Environmentally controlled metabolic chambers were installed within the barn to minimize handling stress, equipped with lighting, daily cleaning, and adequate space. HS was induced using two temperatures (25 °C and 31 °C) and two RH levels: 50% (low RH, LRH) and 80% (high RH, HRH). The cows were exposed to HS for 4 consecutive days, based on previous studies using 3–7 days of exposure in metabolic chambers [14,15]. These environmental conditions were selected to reflect typical summer climates in South Korea, where temperatures range from 25 °C to 31 °C with 50–80% RH [16], while HS typically occurs above 25 °C with 50–60% RH [17]. The metabolic chamber conditions were automatically regulated and recorded at 5 min intervals. Temperature was regulated using an air conditioner (CSVR-Q118E, Carrier Corporation, Seoul, Republic of Korea), while the RH was adjusted with humidifiers (DE-9090UH, Zhongshan Xinhao Electrical, Guangdong, China) and dehumidifiers (EDHA11W3, WINIA, Seoul, Republic of Korea). All early-lactation Holstein cows were provided with a basal diet formulated following the NRC [17] guidelines. The composition of this diet is presented in Table 1. To ensure accurate assessment of HS effects, night-time conditions (19:00–09:00) were maintained at thermoneutral levels to prevent additional stress.

### 2.2. Samplings and Analysis

#### 2.2.1. Dry Matter Intake and Water Intake

Each group was fed twice daily, at 09:00 and 14:00, with water provided five times per day to meet the nutritional needs of early-lactation Holstein cows. DMI and water intake were recorded daily by measuring the feed and water offered at 09:00 and the residual amounts at 08:30 the following morning. Measurements were taken using a GL-6000S scale (G-Tech International Co., Ltd., Uijeongbu, Republic of Korea), with trough weight subtracted to determine net intake. Drinking water was maintained at 15 °C, the optimal temperature for Holstein cows in early lactation. All animals included in this study received a diet formulated in accordance with the NRC [17] guidelines. Feed samples were collected throughout the experimental period and analyzed from the beginning to the end of the study. Samples were oven-dried at 65 °C for 48 h before chemical analysis. Crude protein, ether extract, and ash content were determined using standard AOAC [18] procedures, while neutral detergent fiber and acid detergent fiber were determined according to Van Soest et al. [19].

#### 2.2.2. Milk Yield and Composition

Milking was performed individually for each early-lactating Holstein cow twice daily, at 05:00 and 17:00, using a portable milking machine (PMM 1B EPV170, Italy). Milk yield was measured at each session using a GL-6000S scale (G-Tech International, Uijeongbu-si, Republic of Korea). Milk yield and composition were evaluated over the 7-day experimental period. At each milking session, samples were collected in 50 mL tubes, pooled, and analyzed using a Foss Alle FT1 milk scanner (DK-3400, Hilleroed, Denmark). Energy-corrected milk (ECM) and 3.5% fat-corrected milk (FCM) were calculated as described by Jo et al. [12], while milk energy was determined based on Boerman et al.’s [20] formula, using the following equations:

ECM = (0.327 × milk yield) + (12.95 × milk fat yield) + (7.2 × milk protein yield)

3.5% FCM = (0.4234 × milk yield) + (16.216 × milk fat yield)

Milk energy = milk yield × [(9.29 × milk fat yield + (5.63 × milk protein yield + (3.95 × milk lactose yield)]

Milk yield and composition, including fat, protein, and lactose contents, were averaged over the 3-day adaptation and 4-day HS periods.

#### 2.2.3. Physiological Indicators

Physiological responses were assessed under controlled temperature and RH conditions within the metabolic chambers. Rectal temperature (RT, °C) was measured by inserting a plunge probe (TES 1300, K-Type thermometer, Taipei, Taiwan) into the rectum for 1 min. Measurements were taken at 14:00 on day 3 (adaptation period) and day 7 (HS period). Heart rate (HR, beats per minute) was assessed using a stethoscope.

#### 2.2.4. Hematology, Metabolite, and Hormone Parameters in Blood

Blood samples were collected from the jugular vein at 14:00 on day 3 (adaptation period) and day 7 (HS period) in each group. Ethylenediaminetetraacetic acid-treated vacutainers (Becton–Dickinson, Franklin Lakes, NJ, USA) were used for hematology analysis, which was conducted within 12 h using a VetScan HM2 analyzer (Tampa, FL, USA). For metabolite and hormone analysis, blood was collected using heparinized and nonheparinized vacutainers (BD Vacutainer, Plymouth, UK). Serum and plasma were separated through centrifugation (2000× *g*, 15 min, 4 °C) and stored at −80 °C (U9280-0002, Hamburg, New Brunswick, Canada). Serum samples were analyzed for blood metabolites using an automatic chemical analyzer (7180, Hitachi, Cheonan-si, Republic of Korea). Plasma concentrations of cortisol and haptoglobin were determined using ELISA kits (CUSABIO, Houston, TX, USA; MyBioSource, San Diego, CA, USA) and quantified at 450 nm absorbance with a spectrophotometer (PMT49984, BioTek Instruments, Winooski, VT, USA). The inter- and intra-assay coefficients of variation for cortisol and haptoglobin were 8% and 10%, respectively.

#### 2.2.5. Heat Shock Protein Gene Expression in Hair Follicles

Hair follicle RNA extraction was conducted according to the method described by Kim et al. [21]. Samples were collected from the tails of early-lactation Holstein cows and preserved at 20 °C in RNAlaterTM stabilization solution (LT-02241, Invitrogen, Thermo Fisher Scientific, Lithuania). Following homogenization with an IKA T10 basic homogenizer (Worcester, MA, USA), RNA was extracted for gene expression analysis. RNA purity and concentration were assessed using a NanoDrop 1000 spectrophotometer (Thermo Scientific, Seoul, Republic of Korea), and RNA integrity was confirmed using an Agilent 2100 Bioanalyzer (Agilent, Richardson, TX, USA). Only samples with RNA integrity numbers greater than 6.5 were processed for complementary DNA (cDNA) synthesis. cDNA was synthesized using an iScript cDNA synthesis kit (Bio-Rad, Seoul, Republic of Korea) in a T100 Thermal Cycler (Bio-Rad, Singapore). Quantitative RT-PCR was performed on a CFX Connect Real-Time System (Bio-Rad, USA), following the protocol described by Jo et al. [12]. Primer sequences were designed using the National Center for Biotechnology Information (NCBI) database (Table 2). The gene expression analysis of HSP90 was conducted using CFX Manager 3.1 software (Bio-Rad, USA), and the target gene expression was normalized against glyceraldehyde-3-phosphate dehydrogenase as the reference gene.

### 2.3. Statistical Analysis

Statistical analyses were conducted using a 2 × 2 factorial design in SAS 9.4 (SAS Institute Inc., Cary, NC, USA) and the Mixed procedure. DMI, water intake, and milk yield were analyzed as repeated measures, with early-lactation Holstein cows nested within treatments modeled as random effects. The model was specified as *Y_ijk_ *=* µ *+* α_i_ *+* γ*(*α*)*_ijk_ *+* ε_ijk_*, where *Y_ijk_* is the observed value for cow *k* at sampling day *j* under the treatment *i*; *µ* is the overall mean; *α_i_* is the fixed effect of the treatment (temperature and humidity); *γ*(*α*)*_ijk_* is the random effect of cows nested within the treatment and sampling day; and *ε_ijk_* is the residual error. Covariance structures (compound symmetry, autoregressive order 1, variance components, and unstructured covariance) were assessed, and the structure with the lowest Akaike information criterion was selected [22]. A two-way ANOVA was applied to analyze physiological indicators, milk composition, hematological parameters, metabolites, hormone concentrations, and HSP gene expression in hair follicles. The model was specified as *Y_ijk_ *=* μ *+* α_i_ *+* β_j_ *+* γ_ij_ *+* ε_ijk_*, where *α_i_* and *β_j_* represent the temperature and RH effects, respectively; *γ_ij_* is the temperature and RH interaction; and *ε_ijk_* is the residual error. Tukey’s HSD test was used for post hoc comparisons, given its robustness in controlling errors across multiple group comparisons. Sampling during the adaptation period was included as a covariate, and removed if found to be non-significant. Statistical significance was set at *p* < 0.05, with tendencies noted at *p* < 0.10. Data are reported as means ± standard errors.

## 3. Results

### 3.1. Dry Matter Intake and Water Intake

An increase in RH from LRH to HRH resulted in a decrease in DMI by 16.85% at 25 °C and 7.76% at 31 °C (*p* < 0.05; Table 3). A significant interaction between temperature and RH was also observed for DMI (*p* < 0.05).

### 3.2. Milk Yield and Composition

An increase in RH from LRH to HRH reduced the milk yield by 4.03% at 25 °C and 5.55% at 31 °C (*p* < 0.05; Table 4). At both temperatures, higher RH also significantly decreased milk fat by 14.08% and 26.71%, ECM by 12.14% and 24.45%, 3.5% FCM by 12.01% and 22.38%, and milk energy by 12.45% and 19.93%, respectively (*p* < 0.05). Milk lactose tended to decrease by 4.14% at 25 °C and 3.11% at 31 °C under HRH (*p* = 0.0793).

### 3.3. Physiological Indicators

An increase in RH from LRH to HRH resulted in a 1.51% rise in RT at 25 °C and a 0.05% increase at 31 °C (*p* < 0.05; Table 5). HR showed a tendency to increase by 4.53% at 25 °C and 2.27% at 31 °C under a shift from LRH to HRH conditions (*p* = 0.0894). When the temperature was raised from 25 °C to 31 °C, both RT and HR increased under both LRH and HRH conditions (*p* < 0.05). A significant interaction between temperature and RH was observed for RT (*p* < 0.05).

### 3.4. Blood Profiles

Hematological parameters did not show significant differences by RH (Table 6). A temperature increase from 25 °C to 31 °C resulted in a decrease in granulocyte (GRA) levels and significant increases in mean platelet volume (MPV) and platelet distribution width (PDWc) (*p* < 0.05). Under LRH conditions, red blood cells (RBCs) and hematocrit (HCT) increased when the temperature changed from 25 °C to 31 °C, while both parameters decreased under HRH conditions (*p* < 0.05). Hemoglobin (HGB) increased when the temperature changed from 25 °C to 31 °C under LRH conditions, and decreased under HRH conditions (*p* = 0.0689). Plateletcrit (PCT) tended to decrease as the temperature increased from 25 °C to 31 °C (*p* = 0.0938). GRAs, RBCs, HCT, mean corpuscular hemoglobin (MCH), and mean corpuscular hemoglobin concentration (MCHC) showed significant interactions between temperature and RH (*p* < 0.05). White blood cells (WBCs) showed a significant trend (*p* = 0.0564).

As shown in Table 7, blood metabolite and hormone analysis revealed that glucose concentrations increased by 6.52% at 25 °C and 3.03% at 31 °C as the RH changed from LRH to HRH (*p* < 0.05). Blood urea nitrogen (BUN) concentrations increased by 13.33% at 25 °C and 22.73% at 31 °C, while cortisol concentrations increased by 21.11% and 44.50%, respectively (*p* < 0.05). At LRH, higher temperatures (31 °C) resulted in increased concentrations of glucose, total protein, γ-globulin, inorganic phosphorus (IP), magnesium, gamma-glutamyltranspeptidase (γ-GT), glutamic oxaloacetic transaminase (GOT), and cortisol. Conversely, the concentrations of BUN, albumin, calcium, and cholesterol were lower (*p* < 0.05). Under HRH conditions, glucose, BUN, total protein, albumin, γ-globulin, calcium, IP, magnesium, cholesterol, γ-GT, GOT, and cortisol concentrations were higher at 31 °C than at 25 °C (*p* < 0.05). A significant interaction between temperature and RH was observed for total protein, γ-globulin, calcium, and cholesterol (*p* < 0.05), while albumin (*p* = 0.0568) and magnesium (*p* = 0.0807) showed statistical tendencies.

### 3.5. Heat Shock Protein Gene Expression in Hair Follicles

Analysis of HSP gene expression in hair follicles, using RNA extracted from samples, revealed that HSP90 expression increased by 49.54% at 25 °C and 97.26% at 31 °C when the RH shifted from LRH to HRH (*p* < 0.05; Table 8). Additionally, under LRH and HRH conditions, increasing the temperature from 25 °C to 31 °C resulted in higher HSP90 gene expression in hair follicles (*p* < 0.05).

## 4. Discussion

### 4.1. Dry Matter Intake and Water Intake

HS reduces DMI as the body reduces metabolic heat production, since digestion generates heat. Dairy cows in hot and humid climates show slower growth, highlighting the challenges of these environmental conditions [23]. However, few studies have precisely assessed the impact of RH on DMI in dairy cows. Ruminants depend on evaporative cooling to regulate body temperature [24], but high RH increases HS by slowing moisture loss from the skin, reducing cooling efficiency. Understanding the physiological and biochemical adaptations to HS is important for improving livestock management. Li et al. [25] reported a consistent decline in DMI in goats as the RH increased from 35% to 80% at temperatures of 26 °C, 30 °C, 34 °C, and 38 °C. Similarly, Weniger and Stein [26] found that at 30°C with 60% RH, rumen nutrient decomposition and digestibility decreased by 50% compared to conditions of 35 °C with 50% RH. Consistent with these findings, our study observed a reduction in DMI as RH increased. These findings indicate that elevated RH exacerbates HS, leading to a decrease in DMI as a physiological response to limiting metabolic heat production. A persistent reduction in DMI may negatively impact the dairy industry by compromising production efficiency and animal health.

### 4.2. Milk Yield and Composition

As the ambient temperature rises and the thermal gradient between the animal and its environment decreases, evaporative cooling becomes the primary mechanism for heat dissipation. In cows, this response is most pronounced at 16.6–18.3 °C [27], but its effectiveness diminishes under high RH, which reduces heat loss and increases body temperature [28]. Elevated RH hinders respiratory and skin evaporation, leading to higher body temperature, reduced DMI, and decreased milk yield. Specifically, the milk yield decreased as the RH increased from 20% to 45% at 32 °C [27]. A 25% increase in RH exacerbated HS, impaired heat dissipation, and further decreased DMI and milk yield [29].

An increased core body temperature reduces milk yield and lowers concentrations of milk protein, fat, and lactose [30]. Previous research found that exposure to a THI of 74 for 4 days resulted in reductions in milk fat, milk protein, ECM, 3.5% FCM, and milk energy [31]. A negative correlation (r = −0.39) between RH and milk fat concentration was found [32]. Milk fat reduction is associated with decreased fiber intake and metabolic changes, including elevated insulin concentrations and reduced lipolytic activity [33]. HS also reduces milk protein levels by redirecting amino acids for other tissue needs and gluconeogenesis, while reducing rumen microbial protein synthesis [34]. Although no significant difference was found in milk protein concentration (*p* > 0.05), a trend toward reduction was observed as the RH increased, indicating that a study with a larger sample size may reveal a significant difference. High RH exacerbates these effects by limiting heat dissipation, stimulating the hypothalamus–pituitary–adrenal (HPA) axis, and altering cortisol secretion, which reduces nutrient availability in the mammary gland [35]. Increased amino acid mobilization from muscle and higher glucose consumption reduce the energy availability for milk synthesis. Impaired glucose utilization in peripheral tissues further limits mammary energy supply, suppressing milk protein and fat production [35].

ECM standardizes milk energy content, offering a more precise assessment of dairy cow productivity by accounting for variations in milk fat and protein. This metric reflects metabolic efficiency and energy utilization more effectively than simple milk yield alone. Previous studies have reported a higher ECM in dairy cows without HS across all parity groups [1], with seasonal variations observed—autumn calvers had the highest ECM, while summer calvers had the lowest. A decline in ECM indicates reduced overall energy efficiency or milk yield, whereas a lower FCM value is primarily associated with decreased milk fat content or milk yield. The FCM value adjusts the milk yield based on the fat content, allowing for comparisons across milk with varying fat levels. Reductions in ECM and FCM indicate impaired energy metabolism and nutritional status, likely due to decreased DMI and HS. Milk energy, which represents the total energy from milk fat, protein, and lactose, is also reduced under these conditions. Decreases in ECM, 3.5% FCM, and milk energy can result from lower DMI and increased body temperature, which may suppress mammary cell function [35]. This study found reductions in ECM, 3.5% FCM, and milk energy, indicating that RH-induced HS lowers milk fat, protein, lactose, and yield, thereby reducing milk energy and nutrients. Heat-induced reductions in DMI have been identified as a key factor in milk yield decline. Our study highlights the substantial negative impact of RH on DMI, ECM, 3.5% FCM, and milk energy, underscoring its adverse effects on dairy production.

### 4.3. Physiological Indicators

In dairy cows, the skin plays a vital role in dissipating heat through convection, radiation, conduction, and evaporation. This study excluded heat transfer through solar radiation and surface contact to isolate the effects of other mechanisms. Regulating temperature and RH under HS is essential for welfare and productivity, as it directly influences RT and HR. An increase in body temperature lowers DMI and disrupts digestion, metabolism, and biochemical processes, making RT a reliable indicator of HS.

A higher RH impairs heat dissipation, resulting in heat accumulation and increased RT and HR. While sweating rates may remain consistent across RH levels, higher RH reduces the vapor pressure difference, reducing evaporation cooling efficiency based on thermodynamic principles [36]. Evaporative heat loss through the respiratory tract and mouth is more effective at low-to-moderate RH [37]. In poultry, high RH compromises evaporative cooling, increasing susceptibility to HS [38]. As the RH rises, evaporative and other heat loss mechanisms become less effective, leading to greater heat retention and increased RT under ambient temperatures [39]. In broiler chickens, the RT increased progressively at RH levels of 35%, 60%, and 85% under both 30 °C and 35 °C conditions, showing the effect of RH on thermal regulation [40]. These findings indicate that impaired heat dissipation due to high RH likely contributed to the observed increase in RT.

High RH affects respiratory health by damaging the respiratory epithelium and impairing mucociliary clearance in calves [41]. It also increases disease risk by weakening airway defenses and enhancing pathogen viability. In evaporative cooling systems, excessive RH may exacerbate respiratory stress. Findings from this study confirm that high temperature and RH reduce heat dissipation and increase heat production in early-lactation Holstein cows. An increased HR indicates greater thermoregulatory stress, affecting comfort and lactation, whereas elevated RT reflects severe internal HS. The inhibitory effect of RH on cooling increases the risk of dehydration and heat-related disorders [42]. Improving barn design, ventilation systems, and cooling strategies is essential for protecting cow welfare and sustaining dairy production in humid climates.

### 4.4. Blood Profiles

Blood glucose concentration is a key biomarker of secondary stress, integrating indicators of physiological stress and energy metabolism [43]. Research on the impact of RH on blood metabolites and hormonal regulation in early-lactation Holstein cows remains limited. Ying et al. [43] observed increased glucose concentrations with a rise in RH from 60% to 85%. In this study, higher RH was associated with increased blood glucose concentrations, indicating that excessive RH may exacerbate the metabolic effects of HS by stimulating gluconeogenic activity under adverse conditions [44]. Adrenal hormones are central to glucose regulation, as HS activates the HPA axis, increasing corticosterone secretion under HS, which, in turn, promotes gluconeogenesis and raises blood glucose concentrations [43]. HS also triggers the neuroendocrine system, including the HPA axis, which modulates both metabolic and immune responses [45]. When combined with high RH, HS further intensifies HPA axis activation, increasing cortisol secretion [46], disrupting metabolic balance and immune function, and potentially impairing overall health and lactational performance.

Higher RH intensifies stress responses, leading to increased cortisol secretion under HRH conditions. Lakhani et al. [47] found that Murrah buffaloes showed significantly higher cortisol concentrations at 30 °C with 74% RH compared to 48% RH. Similarly, this study observed a rise in cortisol concentrations with increasing RH, indicating heightened physiological stress. This response is mediated by the sympathetic–adrenal–medullary axis, which is regulated by hypothalamic signaling [48]. Increased cortisol concentrations may enhance glycogenolysis to meet increased energy demands, resulting in higher blood glucose concentrations [43]. Reduced ATP production under HRH conditions may further exacerbate metabolic strains. These findings emphasize the need for effective management strategies to maintain the health and productivity of early-lactation Holstein cows in humid climates. Further studies are needed to elucidate the relationships among RH, cortisol, glycogen, and glucose regulation.

The impact of high RH on urea nitrogen metabolism remains largely unexplored. Urea, the primary byproduct of protein breakdown, enters the gastrointestinal tract, where bacterial urease converts it into ammonia and carbon dioxide. High RH has been shown to significantly reduce erythrocyte Na^+^/K^+^-ATPase activity in mice, with a corresponding negative correlation with plasma BUN concentrations [49]. This enzyme is critical for maintaining membrane potential, cell volume, and neuronal signaling by regulating Na^+^ and K^+^ transport [50]. Impaired Na^+^/K^+^-ATPase function lowers plasma osmolality and erythrocyte sodium concentrations, whereas hyponatremia-induced ADH secretion may enhance urea retention [51]. Elevated plasma BUN concentrations were also observed following 12 h of high-RH exposure [49], which also induced gut microbiota dysbiosis under conditions of 30 °C and 85–90% for 12 h daily [52]. Short-term exposure to very high RH (90% ± 2%) rapidly suppressed Na^+^/K^+^-ATPase activity, reduced plasma osmolality, and increased colonic urea concentrations, potentially altering urea metabolism through microbiota-driven ADH modulation. These findings indicate that elevated RH may increase plasma BUN concentrations through Na^+^/K^+^-ATPase impairment and ADH-mediated mechanisms.

### 4.5. Heat Shock Protein Gene Expression in Hair Follicles

Hair follicle analysis offers a minimally invasive and low-stress method for assessing HSP gene expression in dairy cows, providing an objective measure of physiological stress [21]. HSPs regulate protein structural integrity during HS, with higher expression levels reflecting increased cellular stress. HS induces mRNA transcription and translation of HSPs, particularly HSP90, to protect cells from damage, which explains the increased expression observed in summer. High body temperature and cellular stress activate heat shock factors, which bind to the promoter regions of HSP genes, enhancing their transcription [53]. Under humid conditions, HS—suggested by increased cortisol and BUN concentrations—may contribute to cardiovascular strains, possibly stimulating HSP expression to help mitigate apoptosis and protect heart tissue [54,55]. Alhussien et al. [56] observed that HSP90 expression was significantly higher in tropical dairy cows when exposed to hot–humid conditions (THI = 82) than when subjected to hot–dry conditions (THI = 76). Under hot and humid conditions, HSP90 is rapidly upregulated, enhancing cellular defense by suppressing mitochondrial caspase-9 activation and preserving cytoskeletal integrity. It also interacts with transcription factors to coordinate the expression of additional HSPs and stress-responsive genes, thereby stabilizing myocardial function during thermal humid stress [56,57]. This study provides novel evidence that RH directly affects HSP90 expression in the hair follicles of early-lactation Holstein cows under HS. Further research is needed to clarify the mechanisms underlying HSP response to humid HS by investigating not only HSP90, but also other HS-related markers, such as HSP70, to better understand RH-induced stress.

## 5. Conclusions

Understanding the physiological and productive responses to RH-induced HS will facilitate the development of effective management strategies to mitigate productivity losses in increasingly hot and humid environments. Consistent with previous findings, our study showed that elevated RH significantly reduced DMI, milk yield, and key milk components, including milk fat, ECM, 3.5% FCM, and milk energy, in dairy cows. The decrease in ECM, 3.5% FCM, and milk energy highlights the reduced metabolic energy availability for milk production under humid conditions. Physiological indicators, such as RT, HR, blood glucose, BUN, and cortisol, further confirm the HS response induced by high RH. Moreover, the upregulation of HSP90 gene expression in hair follicles suggests a genetic response to RH-induced HS. Given the substantial impact of RH on dairy productivity, larger-scale studies are needed to clarify its effects, while ventilation-based cooling and RH-targeted monitoring remain essential for maintaining dairy industry resilience under changing climate conditions.

## Figures and Tables

**Table 1:** Chemical composition and amino acid profile of experimental feeds.

Parameters ^1^	TMR	Concentrates
Analyzed values (%, dry matter basis)		
Dry matter	61.91	88.84
Crude protein	6.06	18.06
Crude fat	1.11	2.75
Crude fiber	8.06	6.44
Crude ash	3.15	6.17
Calcium	0.37	0.61
Phosphorus	0.17	0.50
NDF	21.53	21.61
NDIP	2.17	2.82
NDFn	19.36	18.79
ADF	11.30	10.71
ADIP	0.52	0.73
Estimated values ^2^		
NFC	68.15	51.41
tdNFC	71.67	55.27
tdCP	5.46	17.77
tdFA	0.11	1.75

^1^ NDF, neutral detergent fiber; NDIP, neutral detergent insoluble crude protein; NDFn, NDF-NDIP; ADF, acid detergent fiber; ADIP, acid detergent insoluble crude protein. ^2^ Estimated values were calculated based on the NRC (2001) guidelines. NFC, nonfibrous carbohydrate; tdNFC, truly digestible nonfibrous carbohydrate; tdCP, truly digestible crude protein; tdFA, truly digestible fatty acid.

**Table 2:** Primer sequences designed for bovine genes.

Gene	Accession Number ^1^	Sequence (5′ to 3′)	Length (bp)
HSP90	NM_001012670	F: GGAGGATCACTTGGCTGTCA R: GGGATTAGCTCCTCGCAGTT	177
GAPDH	NM_001034034.2	F: GGCAAGGTCATCCCTGAG R: GCAGGTCAGATCCACAACAG	166

HSP90, heat shock protein 90; GAPDH, glyceraldehyde 3-phosphate dehydrogenase. ^1^ Database protein names and accession number: NCBI (http://www.ncbi.nih/gov, accessed on 4 February 2022).

**Table 3:** Effects of relative humidity on productivity under heat stress conditions in lactating Holstein cows.

Temperature (°C)	25		31		SEM	*p*-Value		
RH (%)	LRH	HRH	LRH	HRH		T	RH	T × RH
DMI, kg/day	30.09	25.02	28.87	26.63	0.948	0.0038	0.0169	0.0098
WI, L/day	93.22	99.39	94.88	97.19	2.186	0.8859	0.3861	0.5701

Values are presented as means ± SEM (*n* = 4). LRH, low relative humidity (50%); HRH, high relative humidity (80%); SEM, standard error of the mean; DMI, dry matter intake; WI, water intake; T, temperature; RH, relative humidity.

**Table 4:** Effects of relative humidity on milk characteristics under heat stress conditions in lactating Holstein cows.

Temperature (°C)	25		31		SEM	*p*-Value		
RH (%)	LRH	HRH	LRH	HRH		T	RH	T × RH
Milk yield, kg/d	34.01	32.64	32.82	31.00	0.596	0.1051	0.0091	0.1501
Milk fat, kg/d	1.42	1.22	1.61	1.18	0.046	0.5665	0.0317	0.7017
Milk protein, kg/d	0.94	0.86	0.84	0.79	0.031	0.5063	0.1297	0.3644
Milk lactose, kg/d	1.69	1.62	1.61	1.56	0.050	0.2176	0.0793	0.3012
SNF, kg/d	8.24	8.22	7.91	8.13	0.039	0.7253	0.2429	0.3792
Somatic cells, 1000/mL	118.50	140.17	119.00	56.25	9.205	0.1385	0.4119	0.1357
MUN, mg/dL	13.35	13.20	14.17	14.13	0.421	0.5553	0.1513	0.4523
Acetone, mM	0.09	0.06	0.05	0.03	0.010	0.4740	0.7882	0.5366
BHB, mM	0.11	0.04	0.07	0.08	0.005	0.8849	0.1954	0.2380
Beta-casein, %	2.05	1.93	1.89	2.01	0.023	0.6523	0.1790	0.6862
ECM	37.08	32.58	39.51	29.85	1.089	0.5826	0.0352	0.9668
3.5% FCM	38.12	33.54	41.51	32.22	1.072	0.4789	0.0310	0.9907
Milk energy	25.70	22.50	27.29	21.85	0.702	0.4067	0.0357	0.8445

Values are presented as means ± SEM (*n* = 4). LRH, low relative humidity (50%); HRH, high relative humidity (80%); SEM, standard error of the mean; T, temperature; RH, relative humidity; SNF, solid not fat; MUN, milk urea nitrogen; BHB, beta hydroxybutyrate; ECM, energy-corrected milk; FCM, fat-corrected milk.

**Table 5:** Effects of relative humidity on physiological indicators under heat stress conditions in lactating Holstein cows.

Temperature (°C)	25		31		SEM	*p*-Value		
RH (%)	LRH	HRH	LRH	HRH		T	RH	T × RH
Rectal temperature, °C	38.45	39.03	39.08	39.10	0.060	0.0001	0.0001	0.0001
Heart rate, bpm	82.75	86.50	88.25	90.25	1.218	0.0095	0.0894	0.7343

Values are expressed as means ± SEM (*n* = 4). LRH, low relative humidity (50%); HRH, high relative humidity (80%); SEM, standard error of the mean; T, temperature; RH, relative humidity.

**Table 6:** Effects of relative humidity on blood hematology under heat stress conditions in lactating Holstein cows.

Temperature (°C)	25		31		SEM	*p*-Value		
RH (%)	LRH	HRH	LRH	HRH		T	RH	T × RH
WBC, 10^9^/L	9.10	12.10	9.80	7.43	0.486	0.2647	0.5495	0.0564
LYM, 10^9^/L	4.35	3.12	5.53	3.66	0.394	0.1136	0.6064	0.9054
MON, 10^9^/L	0.29	0.67	0.33	0.45	0.067	0.5946	0.1731	0.4605
GRA, 10^9^/L	4.47	8.32	3.94	3.33	0.421	0.0193	0.1414	0.0476
LYM, %	48.60	25.53	51.63	51.13	3.204	0.1408	0.2670	0.2091
MON, %	3.13	5.15	3.15	5.98	0.615	0.8116	0.1502	0.8227
GRA, %	48.25	69.28	45.23	42.90	3.194	0.1490	0.4499	0.2065
RBC, 10^12^/L	6.37	6.89	6.58	6.23	0.151	0.0229	0.8660	0.0426
HGB, g/dL	9.75	10.30	10.10	9.20	0.177	0.0689	0.8177	0.3334
HCT, %	28.39	30.56	30.27	26.55	0.487	0.0295	0.9104	0.0155
MCV, fL	45.00	44.25	46.50	43.00	0.673	0.9955	0.4240	0.9508
RDWc, %	20.93	19.73	19.70	20.70	0.176	0.4368	0.1796	0.1645
MCH, pg	15.40	14.93	15.45	14.85	0.193	0.5170	0.2918	0.0324
MCHC, g/dL	34.35	33.60	33.33	34.58	0.164	0.6323	0.8578	0.0104
PLT, 100–800 K/uL	634.50	562.50	587.00	498.25	21.211	0.1395	0.8634	0.8438
MPV, fL	7.18	7.05	7.50	7.13	0.069	0.0252	0.1046	0.9611
PCT, %	0.46	0.40	0.45	0.35	0.016	0.0938	0.5531	0.8003
PDWc, %	31.68	31.23	33.80	33.33	0.344	0.0041	0.4564	0.5232

Values are presented as means ± SEM (*n* = 4). LRH, low relative humidity (50%); HRH, high relative humidity (80%); SEM, standard error of the mean; T, temperature; RH, relative humidity; WBC, white blood cell; LYM, lymphocyte, MON, monocyte; GRA, granulocyte; RBC, red blood cell; HGB, hemoglobin; HCT, hematocrit; MCV, mean corpuscular volume; RDWc, red cell distribution width; MCH, mean corpuscular hemoglobin; MCHC, mean corpuscular hemoglobin concentration; PLT, platelet; MPV, mean platelet volume; PCT, plateletcrit; PDWc, platelet distribution width.

**Table 7:** Relationship between relative humidity and blood metabolites and hormones under heat stress conditions in lactating Holstein cows.

Temperature (°C)	25		31		SEM	*p*-Value		
RH (%)	LRH	HRH	LRH	HRH		T	RH	T × RH
Glucose, mg/dL	46.00	49.00	57.75	59.50	1.133	0.0001	0.0156	0.9550
NEFA, uEq/L	231.75	271.50	327.75	251.75	38.929	0.2265	0.4860	0.3346
BUN, mg/dL	11.25	12.75	11.00	13.50	0.420	0.0182	0.0018	0.4168
Total protein, g/dL	6.76	6.40	7.04	7.40	0.088	0.0016	0.5145	0.0366
Albumin, g/dL	3.17	3.12	3.13	3.29	0.036	0.0319	0.7093	0.0568
r-Globulin, g/dL	3.59	3.28	3.92	4.10	0.077	0.0004	0.2807	0.0350
Calcium, mg/dL	8.70	7.90	8.48	9.43	0.122	0.0018	0.6105	0.0017
IP, mg/dL	4.48	4.00	5.80	4.90	0.165	0.0297	0.1053	0.9338
Magnesium, mg/dL	2.08	2.05	2.23	2.38	0.036	0.0072	0.9104	0.0807
Cholesterol, mg/dL	209.50	191.50	181.50	219.75	7.985	0.0019	0.1305	0.0175
r-GT, U/L	12.75	14.00	14.25	18.50	0.572	0.0451	0.2167	0.4563
GOT, U/L	45.00	52.25	54.25	58.50	1.814	0.0040	0.3584	0.6985
Cortisol, ng/mL	124.63	150.94	172.69	249.53	13.879	0.0060	0.0010	0.1290

Values are presented as means ± SEM (*n* = 4). LRH, low relative humidity (50%); HRH, high relative humidity (80%); SEM, standard error of the mean; T, temperature; RH, relative humidity; NEFA, non-esterified fatty acid; BUN, blood urea nitrogen; IP, inorganic phosphorus; r-GT, gamma-glutamyltranspeptidase; GOT, glutamic oxaloacetic transaminase.

**Table 8:** Relationship between relative humidity and heat shock protein gene expression in hair follicles under heat stress (HS) conditions in lactating Holstein cows.

Temperature (°C)	25		31		SEM	*p*-Value		
RH (%)	LRH	HRH	LRH	HRH		T	RH	T × RH
HSP90	1.09	1.63	2.19	4.32	0.258	0.0039	0.0255	0.1522

Values are presented as means ± SEM (*n* = 4). LRH, low relative humidity (50%); HRH, high relative humidity (80%); SEM, standard error of the mean; T, temperature; RH, relative humidity; HSP, heat shock protein.

## Data Availability

The datasets used and/or analyzed during the current study are available from the corresponding author upon reasonable request.

## Abbreviations

The following abbreviations are used in this manuscript:

LRH	Low relative humidity
HRH	High relative humidity
RH	Relative humidity
HS	Heat stress
HSP	Heat shock protein
DMI	Dry matter intake
THI	Temperature–humidity index
T_db_	Dry-bulb temperature
ECM	Energy-corrected milk
FCM	Fat-corrected milk
RT	Rectal temperature
HR	Heart rate
cDNA	Complementary DNA
NCBI	National Center for Biotechnology Information
MUN	Milk urea nitrogen
BHB	Beta-hydroxybutyrate
WBC	White blood cell
LYM	Lymphocyte
MON	Monocyte
GRA	Granulocyte
RBC	Red blood cell
HGB	Hemoglobin
HCT	Hematocrit
MCV	Mean corpuscular volume
RDWc	Red cell distribution width
MCH	Mean corpuscular hemoglobin
MCHC	Mean corpuscular hemoglobin concentration
PLT	Platelet
MPV	Mean platelet volume
PCT	Plateletcrit
PDWc	Platelet distribution width
NEFA	Non-esterified fatty acid
BUN	Blood urea nitrogen
IP	Inorganic phosphorous
r-GT	Gamma-glutamyltranspeptidase
GOT	Glutamic oxaloacetic transaminase
BUN	Blood urea nitrogen
HPA	Hypothalamus–pituitary–adrenal
